# Survival prognostic and recurrence risk factors after single pulmonary metastasectomy

**DOI:** 10.1186/s13019-021-01740-3

**Published:** 2021-12-28

**Authors:** Céline Forster, Amaya Ojanguren, Jean Yannis Perentes, Matthieu Zellweger, Thorsten Krueger, Etienne Abdelnour-Berchtold, Michel Gonzalez

**Affiliations:** 1 Service of Thoracic Surgery, Lausanne University Hospital (CHUV), Rue du Bugnon 46, 1011 Lausanne, Switzerland; 2 Faculty of Biology and Medicine, University of Lausanne (UNIL), Rue du Bugnon 21, 1011 Lausanne, Switzerland

**Keywords:** Pulmonary metastases, Pulmonary metastasectomy, VATS

## Abstract

**Background:**

Identification of the prognostic factors of recurrence and survival after single pulmonary metastasectomy (PM).

**Methods:**

Retrospective analysis of all consecutive patients who underwent PM for a single lung metastasis between 2003 and 2018.

**Results:**

A total of 162 patients with a median age of 64 years underwent single PM. Video-Assisted Thoracic Surgery (VATS) was performed in 83.9% of cases. Surgical resection was achieved by wedge in 73.5%, segmentectomy in 7.4%, lobectomy in 17.9% and pneumonectomy in 1.2% of cases. The median durations of hospital stay and of drainage were 4 days (IQR 3–7) and 1 day (IQR 1–2), respectively. During the follow-up (median 31 months; IQR 15–58), 93 patients (57.4%) presented recurrences and repeated PM could be realized in 35 patients (21.6%) achieved by VATS in 77.1%. Non-colorectal tumour (HR 1.84), age < 70 years (HR 1.77) and previous extra-thoracic metastases (HR 1.61) were identified as prognostic factors of recurrence. Overall survival at 5-year was estimated at 67%. Non-colorectal tumour (HR 2.40) and mediastinal lymph nodes involvement (HR 3.42) were significantly associated with an increased risk of death.

**Conclusions:**

Despite high recurrence rates after PM, surgical resection shows low morbidity rate and acceptable long-term survival, thus should remain the standard treatment for single pulmonary metastases.

*Trial registration*: The Local Ethics Committee approved the study (No. 2019–02,474) and individual consent was waived.

## Background

Pulmonary metastases occur in 30–40% of patients with known solid cancer and their appearance indicates a progression of the primary tumour, thus a worsening of the prognosis [1]. Surgical resection of pulmonary metastases may be considered a valid part of the multimodal treatment in patients who have no other distant metastases and can tolerate complete resection of the metastases [2]. In addition to local disease control, surgery allows confirmation of the diagnosis by providing suitable metastatic tissue for histopathological analysis. This facilitates more personalized subsequent systemic treatments, such as targeted therapy or immunotherapy [3].

Patients presenting only a single pulmonary metastasis represent 45–75% of all patients with pulmonary metastases [4, 5]. Single pulmonary metastases are generally managed surgically with a curative intent, but recently there has been a growing interest in various non-surgical approaches, such as radiofrequency or stereotaxic radiotherapy, as promising alternatives in terms of local control [6]. Complete resection of solitary metastases is generally achieved by non-anatomical wedge resection when they are in peripheral locations. Anatomical resections (segmentectomy or lobectomy) may be an alternative for larger or central lesions. Minimally invasive approaches are gaining acceptance in pulmonary metastasis patients, leading to lower post-operative morbidity, shorter duration of hospital stay and oncological outcomes equivalent to those of open thoracotomy [7, 8]. Patients with single pulmonary metastases have been reported to present a better prognosis and an increased overall survival in comparison to patients bearing multiple pulmonary metastases [5, 9–11]. However, little is known about the recurrence rate and risk factors in this specific group of patients. Because they represent a non-negligible sub-sample of all PM patients, and because the specific configuration of a single lung metastasis sets these patients apart in terms of possible surgical cure of their disease, we herewith propose to focus on them only.

The aim of this study was to analyze the surgical outcomes of patients operated for a single pulmonary metastasis. We also evaluated the risk factors for recurrence and identified prognostic factors of shorter survival.

## Methods

### Patient selection and study design

This retrospective study reviewed all consecutive patients with a single pulmonary metastasis who underwent a surgical pulmonary metastasectomy (PM) with curative intent in our institution between July 2003 and November 2018. Patients undergoing diagnostic procedures only and non-surgical patients were excluded. A total of 264 patients were treated in the period of interest, of which 162 had a single pulmonary metastasis. Pre-operatively, all patients were discussed by an interdisciplinary tumour board to assess the criteria, which had to be fulfilled for PM [2]. These are: (1) the patient can withstand surgical intervention; (2) the primary tumour is controlled; (3) there is no other extra-pulmonary metastases or if there is, it can be resected completely before PM; (4) the pulmonary metastasis can be completely resected without impacting the patient’s respiratory functions; (5) there is no other alternative systemic treatment. All patients underwent a thin-slice (1 mm) helical chest CT-scan within 30 days before surgery, which was reviewed by a senior radiologist. Moreover, a Positron Emission Tomography (PET) scan was realized when the primary tumour showed high uptake of Fluorodeoxy-Glucose (FDG) to exclude extra-thoracic metastases. Pre-operative diagnosis by bronchoscopy or percutaneous biopsy under CT-guidance was individually discussed but not mandatory.

### Data collection

We retrospectively extracted the data from our electronic database and reviewed medical records. Data included: patient’s demographics; comorbidities; primary tumour and pulmonary metastasis histology; surgical characteristics; post-operative outcomes up to 30 days after surgery; recurrences, if any, and their localisation; repeated PM characteristics. In addition, disease-free interval (DFI) and overall survival (OS) were assessed. The DFI1 was defined as the interval between the primary tumour resection and the first PM and the DFI2 was defined as the interval between first PM and cancer recurrence. OS was defined as the percentage of patients alive on November 2019, when the follow-up was completed or date of last news. Pulmonary metastases that were diagnosed at the same time as the primary tumour diagnosis were defined as synchronous metastases.

The Local Ethics Committee approved the study (No. 2019-02474) and individual consent was waived.

### Operation and follow-up

The PM was performed under general anaesthesia with single-lung ventilation. If necessary, a percutaneous hook wire device was inserted under CT-scan guidance in the pre-operative phase to facilitate the intraoperative detection of the metastasis. The surgical approach (open or thoracoscopy) and the extent of pulmonary resection (wedge or anatomical resection) were individually discussed for all patients based on the metastasis characteristics (size, localisation). Parenchyma-sparing resection (wedge) and minimally-invasive approach were preferred when feasible. Our Video-Assisted Thoracic Surgery (VATS) approach consisted in a classical three-port anterior approach and the thoracotomy was a standard posterolateral incision in the fifth intercostal space. Lymph node involvement was not considered a contra-indication for surgery. However, lymph node dissection was only realized for lesions of more than 2 cm in diameter, centrally located, requiring an anatomical resection, or when lymph node involvement was suspected on pre-operative radiological exams. All specimens were extracted in a protective bag. The specimens were then examined by the operator in order to assess the completeness of resection. In case of doubt, a histological frozen section was performed. After surgery, all cases were discussed once again in the interdisciplinary tumour board to evaluate the indications for an adjuvant therapy. The follow-up consisted in a thoraco-abdominal CT-scan at 3, 6, 12, 18 and 24 months and then on a yearly basis.

### Statistical analysis

Binary variables are expressed as percentages and continuous variables are presented as median with interquartile range (IQR). OS and PFS were calculated using the Kaplan–Meier and log-rank analyses. Cox regression for uni- and multivariable analyses were applied to identify prognostic factors of recurrence and survival. A two-tailed hypothesis was used and significance accepted if *p* < 0.05. The statistical analysis was performed using the Stata version 14 software (StataCorp, Texas USA).

## Results

Single PM was performed in a total of 162 patients (female/male: 72/90). Patient characteristics are shown in Table 1. The primary tumour origin included colorectal carcinoma (31.5%), melanoma (20.4%), sarcoma (14.8%) and other origins (33.3%). Pulmonary metastases were synchronous in 14.2% and metachronous in 85.8% of cases. In case of metachronous metastases, the median DFI1 was 22.5 months (IQR 10–47 months). Fifty patients (30.9%) had been previously treated by radiotherapy or surgery for another metastases, with 36% of cases in the liver, 28% in local lymph nodes, 6% in the brain and 30% in other localisations. Chemotherapy was administered in 75 patients (46.3%) before the first PM.

**Table 1:** Patient characteristics and surgical characteristics of first single pulmonary metastasectomy (PM)

Single PM	162
Sex	
Female	72 (44.4%)
Male	90 (55.6%)
Age (median)	64 [IQR 55–71]
Comorbidities	
Cardiopathy	12 (7.4%)
High blood pressure	61 (37.7%)
Pulmonary disease	13 (8%)
Tobacco exposure	49 (30.3%)
Diabetes	23 (14.2%)
Renal failure	11 (6.8%)
Immunosuppression	6 (3.7%)
Primary tumour	
Colorectal	51 (31.5%)
Melanoma	33 (20.4%)
Sarcoma	24 (14.8%)
Other	54 (33.3%)
Pulmonary metastasis	
Size [mm] (median)	10.5 [7–16]
Margins [mm] (median)	6 [3–12]
R0	159 (98.2%)
R1	3 (1.9%)
Lymph node involvement	9 (5.6%)
First PM	
VATS	136 (83.9%)
Thoracotomy	26 (16.1%)
Wedge resection	119 (73.5%)
Segmentectomy	12 (7.4%)
Lobectomy	29 (17.9%)
Pneumonectomy	2 (1.2%)
Mediastinal lymph nodes dissection	36 (22.2%)
Post-operative outcomes	
Overall 30-d mortality	0
Overall 30-d morbidity	19 (11.7%)
Pulmonary complications	12 (7.4%)
Cardiac complications	3 (1.9%)
Duration of drainage [days] (median)	1 [IQR 1–2]
Duration of hospital stay [days] (median)	4 [IQR 3–7]
Readmission (30-d)	2 (1.2%)
Re-operation (30-d)	1 (0.6%)

*PM* pulmonary metastasectomy, cardiopathy (defined as the presence of ischemic events in the past, cardiac insufficiency, arrhythmia or aortic aneurysm), high blood pressure (defined as systolic arterial pressure > 140 mmHg), pulmonary disease (defined as the presence of chronic obstructive pulmonary disease, fibrosis, pulmonary hypertension or sleep apnoea syndrome), diabetes (defined as fasting plasma glucose > 7 mmol/l), renal failure (defined as glomerular filtration rate < 30 ml/min/1.73 m^2^). R0 (defined as the absence of cancer cells seen microscopically at the tumor site), R1 (defined as the presence of cancer cells microscopically at the tumor site), R2 (defined as macroscopic residual tumor at cancer site or regional lymph nodes)

A VATS was performed in 136 patients (83.9%) and open thoracotomy in 26 patients (16.1%). There was one case of conversion (accounted for in the latter group) because of a centrally located lesion non-resectable by VATS. Surgical resection was achieved by wedge in 73.5%, segmentectomy in 7.4%, lobectomy in 17.9% and pneumonectomy in 1.2% of cases. Mediastinal lymph node dissection was performed in 36 patients (22.2%). The median durations of drainage and of hospital stay were 1 day (IQR 1–2) and 4 days (IQR 3–7), respectively. The overall post-operative complication rate at 30 days was 11.7%, with 7.4% of pulmonary and 1.9% of cardiac complications. During the 30 post-operative days (POD) period, two patients (1.2%) were readmitted. The first one presented an inflammatory lung effusion which was treated by drainage. The second one had positive histopathological margins and the resection was therefore completed. There was no 30-day mortality.

**Fig. 1:**
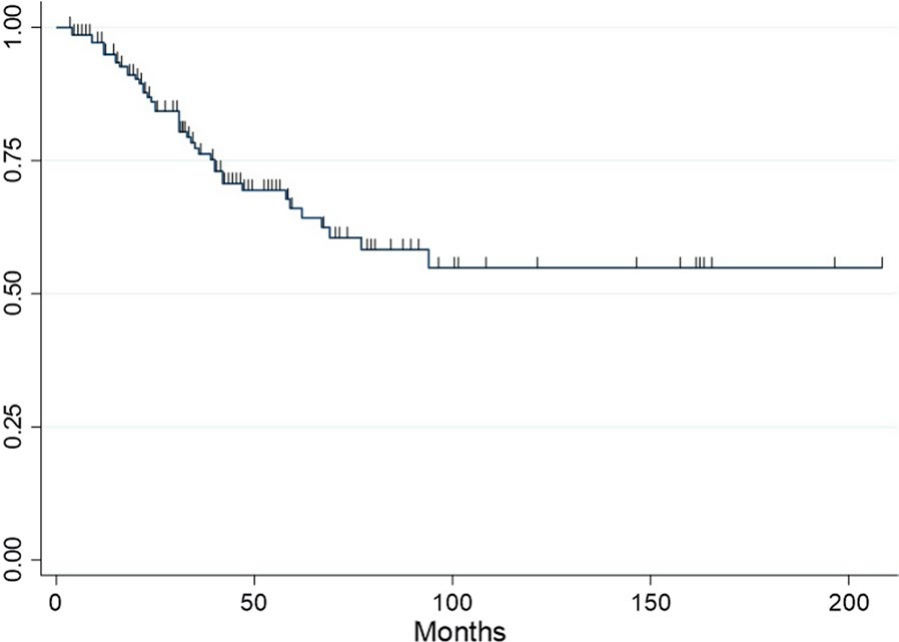
Kaplan–Meier curve of overall survival (OS) after first single pulmonary metastasectomy (PM)

The median follow-up time was 31.5 months (IQR 15–58). Recurrences were observed in 93 patients (57.4%), with 13% in the lung only, 16.1% distantly and 28.4% in both localisations (Table 2). Lung recurrences were ipsilateral in 50.8% of cases, contralateral in 31.3% and bilateral in 17.9% of cases. The median DFI2 was 11 months (IQR 3–31). Thirty-five patients (21.6%) underwent repeated PM (RPM), by VATS in 77.1% and wedge resection in 77.1% of cases. The 5-year OS was 67% after first PM (Fig. 1).

**Table 2:** Recurrence characteristics

	Number of patients (percentage)
Recurrence	93 (57.4%)
Lung only	21 (13%)
Distant	26 (16.1%)
Both lung and distant	46 (28.4%)
Ipsilateral	34 (50.8%)
Controlateral	21 (31.3%)
Bilateral	12 (17.9%)
Repeated pulmonary metastasectomy	35 (21.6%)
VATS	27 (77.1%)
Thoracotomy	9 (25.7%)
Wedge resection	27 (77.1%)
Segmentectomy	6 (17.1%)
Lobectomy	6 (17.1%)
Pneumonectomy	1 (2.9%)
Mediastinal lymph nodes dissection	8 (22.9%)

*VATS* video-assisted thoracic surgery

Several prognostic factors of recurrence after PM were identified by uni- and multivariable analysis. The factors found to be significant after multivariable analysis were: age < 70 years (HR 1.77, 95% CI 1.06–2.96, *p* = 0.03); non-colorectal tumour (HR 1.84, 95% CI 1.14–2.96), *p* = 0.01); presence of prior extra-thoracic metastases (HR 1.61, 95% CI 1.05–2.47, *p* = 0.03) (Table 3). Similarly, two factors were associated with an increased risk of death: non-colorectal tumour (HR 2.40, 95% CI 1.11–5.22, *p* = 0.03) and mediastinal lymph nodes involvement (HR 3.42, 95% CI 1.03–11.41, *p* = 0.04) (Table 4).

**Table 3:** Uni- and multivariate analyses of prognostic factors of recurrence after single pulmonary metastasectomy (PM)

Variables	Univariate		Multivariate	
	HR (95% CI)	*p* value	HR (95% CI)	*p* value
Female sex	1.02 (0.67–1.55)	0.92		
Age < 70 years	1.84 (1.1–3.06)	0.02	1.77 (1.06–2.96)	0.03
Non-colorectal tumour	1.76 (1.09–2.84)	0.02	1.84 (1.14–2.96)	0.01
Previous extra-thoracic metastases	1.65 (1.08–2.54)	0.02	1.61 (1.05–2.47)	0.03
Chemotherapy before first PM	0.84 (0.55–1.28)	0.42		
DFI1 < 12 months	1.29 (0.81–2.02)	0.28		
Synchronous metastasis	1.42 (0.77–2.63)	0.26		
VATS	1.15 (0.61–2.17)	0.66		
Wedge resection	1.26 (0.77–2.09)	0.35		
Margins of the pulmonary metastasis < 5 mm	1.13 (0.75–1.72)	0.54		
Size of the pulmonary metastasis < 20 mm	0.91 (0.53–1.57)	0.74		
Mediastinal lymph nodes involvement	1.56 (0.57–4.29)	0.39		

*HR* hazard ratio, *CI* confidence interval, *PM* pulmonary metastasectomy, *DFI1* disease-free interval (defined as the interval between primary tumour resection and first PM), *VATS* Video-Assisted Thoracic Surgery

**Table 4:** Uni- and multivariate analyses of prognostic factors of worse survival after single pulmonary metastasectomy (PM)

Variables	Univariate		Multivariate	
	HR (95% CI)	*p* value	HR (95% CI)	*p* value
Female sex	1.27 (0.69–2.34)	0.45		
Age > 70 years	0.83 (0.39–1.75)	0.62		
Non-colorectal tumour	2.44 (1.12–5.29)	0.02	2.40 (1.11–5.22)	0.03
Previous extra-thoracic metastases	0.81 (0.42–1.56)	0.55		
Chemotherapy before first PM	1.00 (0.54–1.85)	0.99		
DFI1 < 12 months	1.32 (0.66–2.65)	0.43		
Synchronous metastasis	1.97 (0.82–4.70)	0.13		
VATS	1.04 (0.44–2.48)	0.93		
Wedge resection	0.93 (0.47–1.86)	0.84		
Margins of the pulmonary metastasis < 5 mm	0.80 (0.43–1.49)	0.48		
Size of the pulmonary metastasis < 20 mm	0.96 (0.43–2.18)	0.93		
Mediastinal lymph nodes involvement	3.60 (1.08–12.03)	0.04	3.42 (1.03–11.41)	0.04

*HR* hazard ratio, *CI* confidence interval, *PM* pulmonary metastasectomy, *DFI1* disease-free interval (defined as the interval between primary tumour resection and first PM), *VATS* Video-Assisted Thoracic Surgery

## Discussion

We report on a large series of 162 patients with a single pulmonary metastasis who underwent a PM from 2003 to 2018. Our data indicates that these patients present favourable overall survival (median 31.5 months; 5-year OS of 67%). However, a majority of patients (57.4%) developed recurrences with a short median DFI2 (11 months) after PM.

A PM is generally proposed when the following selection criteria are met: a controlled primary tumour, no extra-thoracic or mediastinal lymph node metastatic spread and sufficient pulmonary reserves to tolerate the resection of all identified metastases [2]. The improvement of surgical techniques and radiological imaging and the recent advances in systemic therapies with the development of new chemotherapeutic agents have contributed to an increase in the numbers of PM procedures. Patients with solid tumours frequently present a single pulmonary nodule, which may not necessarily be a metastasis. Indeed, pulmonary metastases may radiologically resemble other conditions, such as primary lung cancers or benign inflammatory lesions. Surgical resection is sometimes the only way to histologically confirm or infirm the diagnosis of a metastatic disease. Interestingly, in a recent series of cancer patients, VATS resection of solitary nodules allowed the diagnosis of metastases in only 50% of cases [3]. This point is particularly relevant in the context of the development of non-surgical therapies, such as stereotaxic radiotherapy or radiological ablative techniques, where histological diagnosis is rarely reported.

In surgical series, the presence of a solitary metastasis is a frequent situation and represents 47% to 70% of all pulmonary metastatic cases [5, 12]. Many studies have reported that the number of pulmonary metastases is a prognostic factor of survival [5, 13–17]. In colorectal cancer patients, a recent large systematic review including 8361 patients undergoing PM reported that the isolated unilateral lung metastases represented a favourable prognostic factor [12]. A meta-analysis including more than 20 studies showed an increased risk of death (HR 2.04) for multiple lung metastases [9]. In a large series of 615 patients with colorectal cancer, Cho et al. also demonstrated that the number of pulmonary metastases directly influenced the survival, with an overall 5-year survival rate of 70% in the subgroup with single pulmonary metastases compared to 56.2% in the subgroup with 2–3 metastases (*p* < 0.001) [10]. Similar results with better survival for patients with a solitary pulmonary metastasis were also described for other primary tumours: sarcoma (HR 1.16, 95% CI 1.10–2.503, *p* = 0.016), melanoma (HR 1.4, 95% CI 1.1–1.7, *p* = 0.013) and renal cell carcinoma (HR 1.55, 95% CI 1.18–2.03, *p* = 0.002) [13, 18, 19].

In our study, we decided to focus on the population of patients bearing one pulmonary metastasis only because this population represents a non-negligible fraction of all pulmonary metastatic patients, and because it is by definition most amenable to surgical cure of its disease. Our aim was to evaluate risk factors for recurrence and risk factors for worse survival specifically for this population. Most of the PM were performed by VATS (84%). Pulmonary metastases are generally peripheral and small-sized (median diameter: 10 mm), making them easily accessible for a non-anatomical resection by VATS. However, some surgeons still perform thoracotomies in order to palpate the lung and identify other lesions. Nowadays, this paradigm is changing for solitary metastases and VATS approach is becoming the preferred approach, as showed in a survey of cardiothoracic surgeons in Great Britain and Ireland reporting that VATS was used by 85% of surgeons in case of isolated pulmonary metastatic lesions [20]. Indeed, the 1-mm thin-slice CT-scans are very sensitive and can detect nodules of less than 5 mm in diameter, making the bimanual palpation obsolete.

The concordance between radiological imaging and pathological findings was analyzed by the Spanish prospective registry of PM [21]. In this study, solitary nodules were present in 73% of colorectal cancer patients who underwent thoracotomy with bimanual palpation of the lung. The radiological and pathological agreement was 95%. In another series, only 7% of patients with single nodule on pre-operative CT-scan presented more metastatic lesions on pathological analysis after resection by thoracotomy [22]. Thus, these recent results suggest that VATS is a valid approach, at least for patients with a single lesion on pre-operative imaging. In our study, the Cox regression analysis did not find any correlation between the surgical approach (VATS vs. thoracotomy) and the increased risk of recurrence (HR 1.15, 95% CI 0.61–2.17, *p* = 0.66).

Wedge resections using staplers accounted for 74.1% of cases. Anatomical resections (segmentectomy or lobectomy) were reserved for centrally located or larger lesions, the objective being to achieve safe margins, a result known to improve prognosis [23]. We did not find any association between the type of surgical resection and the survival prognosis (HR 0.93, 95% CI 0.44–2.48, *p* = 0.93). Major resections could be justified for selected patients with larger or centrally located pulmonary metastases with favorable results, as demonstrated in a multicenter prospective study reporting an increased survival rate in comparison with non-anatomical resections for colorectal cancer patients with pulmonary metastases (55 vs. 28.3 months) [24].

The post-operative outcomes were favourable with an overall low morbidity of 11.7% with minor complications and a 30-day mortality rate of 0%. These results are consistent with other surgical series about pulmonary metastases [25, 26].

Despite our expectation to observe better prognosis for patients bearing solitary nodules, we observed a recurrence rate of 57.4% in that population. We identified three prognostic factors of recurrence: age under 70 years (HR 1.77, 95% CI 1.06–2.96, *p* = 0.03), prior treatment of extra-thoracic metastases (HR 1.61, 95% CI 1.05–2.47, *p* = 0.03) and non-colorectal origin (HR 1.84, 95% CI 1.14–2.96, *p* = 0.01). These elements are generally correlated with a biologically aggressive behaviour of the primary tumour, which are more prone to generating recurrences.

In our study, of the 93 (57.4%) patients who presented a recurrence, only 35 (21.6%) fulfilled the criteria to undergo an RPM, due to the invasion of other organs or poor residual lung capacity. Our indications for redo surgery are identical to the indications for initial surgery. Interestingly, most of the RPM have been performed by VATS (77.1%) and by wedge resection (77.1%). We observed that VATS procedures induced fewer adhesions and chest wall sequelae than thoracotomies. Thus, repeated VATS procedures were easier to perform.

The 5-year overall survival rate of single PM was 67%, which compares favourably with data from recent literature. With our results, we could identify two factors predictive of shortened survival: non-colorectal tumour origin (HR 2.40, 95% CI 1.11–5.22, *p* = 0.03) and mediastinal lymph nodes involvement (HR 3.42, 95% CI 1.03–11.41, *p* = 0.04). The primary tumour origin has been shown to influence survival with better survival rates in epithelial cancers than in sarcomas or melanomas [5]. Hirai et al. showed that colorectal cancer patients had a better survival rate than patients with other primary organs involved (*p* = 0.003) [4]. In our study, we chose to analyse only two subgroups of primary tumours (colorectal and non-colorectal) because of the high frequency of colorectal tumours (31.5%) compared to other types. The non-colorectal subgroup included melanoma, sarcoma, and others (germ cell, head and neck, breast, urological, gynaecological, thyroid and other).

Many studies have reported a long DFI as being a favourable prognostic factor of survival [5, 13]. In a recent meta-analysis of renal cancer patients with pulmonary metastases, both the synchronous metastases and a short DFI were associated with poor survival rates [13]. In our study, a DFI1 < 12 months did not have any correlation with prognosis.

Lymph node dissection was realized in 22.2% of cases and only nine patients (5.6%) presented hilar or mediastinal lymph node involvement, which was correlated to worse survival rate (HR 3.42, 95% CI 1.03–11.41, *p* = 0.04). While survival of pulmonary metastatic patients is affected by metastatic invasion of the lymph nodes, it remains unclear if systematic lymph node dissection during solitary PM brings any benefits in terms of local recurrence or survival [1]. Lymph node dissection was not performed routinely for solitary peripheral lesions and was reserved for centrally located or larger lesions requiring an anatomical resection. Our rate of lymph node dissection is relatively low in comparison with other series, but does not seem to correlate to the survival rate in this group of patients and in the timeframe that we studied.

Our study presents several limitations, the first one being the retrospective single-center design with a small collective of patients. Next, our study included only patients who underwent a surgical resection of their single pulmonary metastasis. Other patients with a single pulmonary metastasis who were not treated by surgery were not included, thus creating a selection bias. However, our selection criteria mentioned this factor and 5-year survival and other outcomes were described only for included patients, namely those who underwent surgery. Another limitation is the variety of primary tumor types, thus inducing a heterogeneity of the studied population. However, we selected only the patients with a single pulmonary metastasis and reported the primary tumor types, which we stratified along clear lines. Moreover, the patients were included over a 15-year period, thus smoothing out influences on the prognosis that might be due to the evolution of systemic and radiologic therapies. It should be noted also that the exact 5-year survival figures could only be calculated on a fraction of the patient population (those treated until 2015).

## Conclusions

In conclusion, our results demonstrate that surgical resection of a single pulmonary metastasis is beneficial for the patients, thanks to low post-operative morbidity and mortality rates, as well as to an acceptable survival duration. The VATS approach should be preferred when feasible, owing to its low morbidity and mortality rates. However, local or distant recurrences are frequent and RPM can be achieved in a substantial number of cases, especially when the initial PM was carried out by VATS. We found risk factors for recurrence to be a younger age (< 70), primary tumour of non-colorectal origin and a history of extra-thoracic metastases. Similarly, risk factors for worse survival were found to be a primary tumour of non-colorectal origin and invasion of the mediastinal lymph nodes.

## Data Availability

The datasets used and/or analyzed during the current study are available from the corresponding author on reasonable request.

## Abbreviations

PM: Pulmonary metastasectomy
VATS: Video-assisted thoracic surgery
HR: Hazard ratio
PET: Positron emission tomography
FDG: Fluorodeoxy-glucose
DFI1: Disease-free interval 1 (defined as the interval between primary tumour resection and first PM)
DFI2: Disease-free interval 2 (defined as the interval between first PM and cancer recurrence)
OS: Overall survival
IQR: Interquartile range
RPM: Repeated pulmonary metastasectomy
CI: Confidence interval
